# Extracellular Vesicles in Non-alcoholic Fatty Liver Disease and Alcoholic Liver Disease

**DOI:** 10.3389/fphys.2021.707429

**Published:** 2021-07-14

**Authors:** Dongqing Wu, Huaqing Zhu, Hua Wang

**Affiliations:** ^1^Laboratory of Molecular Biology, Department of Biochemistry, Anhui Medical University, Hefei, China; ^2^Department of Oncology, The First Affiliated Hospital of Anhui Medical University, Hefei, China; ^3^Inflammation and Immune Mediated Diseases Laboratory of Anhui Province, Anhui Medical University, Hefei, China

**Keywords:** extracellular vesicles, non-alcoholic fatty liver disease, alcoholic liver disease, pathogenesis, biomarker

## Abstract

As the largest vital solid organ in the body, liver is consisting of multiple types of cells including hepatocytes, Kupffer cell, hepatic stellate cells (HSCs), liver sinusoidal endothelial cells (LSECs), and other immune cells. The communication between these cells is critical in maintaining liver function homeostasis, and dysregulation of such communication contributes to the pathogenesis of various liver diseases. Extracellular vesicles (EVs), including exosomes and ectosomes, act as important mediators of cell-to-cell communication. EVs can be produced and uptaken by a wide range of cells including all types of cells in the liver. Growing evidences show that EVs are involved in the development of liver diseases, especially non-alcoholic fatty liver disease (NAFLD) and alcoholic liver disease (ALD). In this review, we will summarize recent advance in how EVs production are altered in NAFLD and ALD and how the changes of EVs quantity and cargos influence the progression of these diseases. The therapeutic and diagnostic potential of EVs in NAFLD and ALD will be also discussed in this review.

## Introduction

The term extracellular vesicles (EVs) refers to a highly heterogeneous population of cell-derived membrane-enclosed structures (van Niel et al., 2018). EVs can be generally divided into two classes, ectosomes and exosomes (Gurunathan et al., 2021). They are presenting in all biological fluids such as blood, milk, saliva, intercellular fluid and play pleiotropic roles in cell-to-cell communications (Ratajczak and Ratajczak, 2020). EVs carry various cargoes of proteins, lipids, and genetic materials that can deliver signals to induce physiological or pathological changes in recipient cells (Cocozza et al., 2020).

In the field of liver diseases, the number of publications about EVs has grown exponentially over past decade. EVs are emerging as key players in the pathogenesis and progression of metabolic liver diseases including non-alcoholic fatty liver disease (NAFLD) and alcoholic liver disease (ALD; Eguchi and Feldstein, 2018). NAFLD and ALD are serious health issues around the world. They can progress to hepatocyte injury, steatohepatitis, or cirrhosis, an entity designated as alcoholic steatohepatitis (ASH) and non-alcoholic steatohepatitis (NASH; Mitra et al., 2020). In these diseases, a large number of EVs are released by stressed/damaged hepatocytes and act on the target cells to promote inflammation, angiogenesis, and fibrosis (Hernandez et al., 2020). Additionally, as an effective way to deliver protein or nuclear acid to target cells, EVs have been considered as a potential tool for the therapy of liver diseases including NAFLD and ALD. They also have been suggested as diagnostic and prognostic biomarkers for liver diseases (Szabo and Momen-Heravi, 2017; Urban et al., 2019; Wiklander et al., 2019; Thietart and Rautou, 2020).

Here, we focus on the current state of knowledge pertaining to EVs, including how EVs production is regulated in NAFLD and ALD and how EVs mediated intercellular communications contribute to the pathogenesis and progression of NAFLD/ALD. We will also discuss possible therapeutic and diagnostic applications of EVs in NAFLD and ALD.

## The Biogenesis, Cargos, and Biologic Function of EVs

### The Biogenesis of EVs

**Figure 1:**
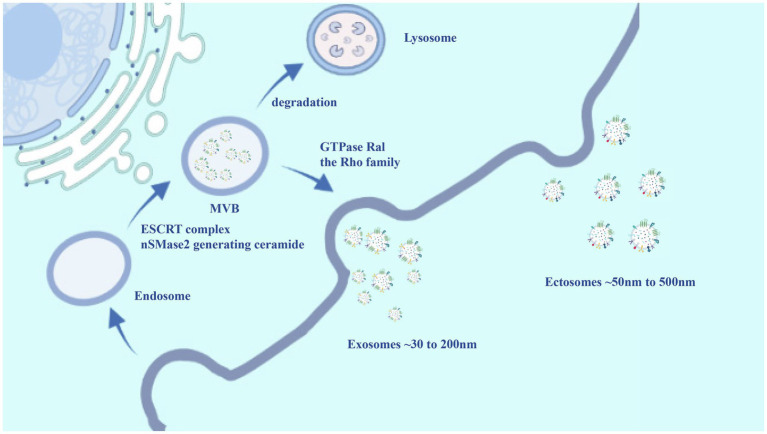
The biogenesis of extracellular vesicles (EVs). Exosomes and ectosomes are two types of EVs secreted by cells. Exosomes (~30–200 nm in diameter) originate from the endosomal pathway and are secreted by the fusion of multivesicular bodies (MVBs) with the plasma membrane. Ectosomes (size range ~50–500 nm) are generated by the direct outward budding of the plasma membrane. The budding of EVs involves a variety of mechanisms, and is a combination of one or more of the following mechanisms: endosomal sorting complexes required for transport (ESCRT) complex, neutral sphingomyelinase 2 (nSMase2) generating ceramide, GTPase Ral, and the Rho family. However, specific biogenesis mechanisms are not yet clearly known. EVs are highly heterogeneous population. The cargos of EVs contain different types of cell surface proteins, intracellular protein, genetic materials [such as DNAs, messenger RNAs (mRNAs), and miRNAs], and lipid.

Extracellular vesicles are membrane-enclosed nanostructures, they can be secreted by specific tissues and cells *in vitro* and *in vivo*. For example, all types of cells in the liver have been proved to generate EVs under *in vitro* conditions (Xie et al., 2019). Based on their biogenesis mechanisms, two major categories of EVs have been described: Ectosomes and Exosomes (Figure 1). Exosomes are approximately 30–200 nm in diameter (Pegtel and Gould, 2019). They arise from endosomal multivesicular bodies (MVBs), which fuse with the plasma membrane and then release into extracellular space and circulation (Doyle and Wang, 2019). During this process, early endosomes are formed by the inward budding of the plasma membrane, or in some cases from the trans-Golgi network (Teng and Fussenegger, 2020). Early endosomes fuse to form late endosomes and finally generate MVBs. Exosomes are essentially intraluminal vesicles (ILVs) of MVBs. Multiple molecular mechanisms of ILVs generation in MVBs have been revealed. Furthermore, accumulating evidences supports that the endosomal sorting complexes required for transport (ESCRTs) play a critical role in the biogenesis of MVBs and cargo sorting (Sun et al., 2018). ESCRTs consist of four complexes of protein components, including ESCRT-0, ESCRT-I, ESCRT-II, and ESCRT-III (Mashouri et al., 2019). The ESCRT-0 components recognizes and sequesters ubiquitylated cargos in the endosomal membrane (Juan and Furthauer, 2018), ESCRT-I/II complexes appear to be responsible for membrane deformation into buds with sequestered cargo (Frankel and Audhya, 2018), and then ESCRT-III subcomplexes drive the fission of vesicle (Larios et al., 2020). In addition to the ESCRTs mechanism, the biogenesis of MVBs depends on other mechanisms. For instance, neutral sphingomyelinase 2 (nSMase2) can regulate the production of bioactive lipid ceramide, thereby triggering the maturation of MVBs (Kosaka et al., 2010; Shamseddine et al., 2015). Eventually, exosomes are formed in the extracellular space by fusing MVBs with the plasma membrane and releasing the contained ILVs. The small GTPase Ral such as Rab27 a/b, Rab35, and RalA has been widely accepted to participate in the progression (Maas et al., 2017). Specifically, some proteins also are involved in the biogenesis of exosomes, and they can be used as markers for exosomes such as CD9, CD81, CD63, flotillin, TSG101, ceramide, and Alix (Kalluri and LeBleu, 2020).

By contrast, ectosomes with a size range of ~50–500 nm in diameter that generated by the direct outward budding of the plasma membrane (Gurunathan et al., 2021). They are also known as microparticles or microvesicles. Less is known about the molecular mechanisms of ectosome biogenesis compared with that of exosomes. Recent evidence demonstrates that two ESCRT subcomplexes may be involved in the outward-budding of ectosomes. The ESCRT-I component TSG101 is recruited to the cell surface by arresting domain-containing protein 1 (ARRDC1), directly causes the evagination of plasma membrane and the release of ectosomes (Nabhan et al., 2012). The negative curvature-promoting ESCRT-III proteins could mediate plasma membrane deformation and release of ectosomes (Chiaruttini et al., 2015). Additional mechanisms that regulate the biogenesis of ectosomes include the small GTPase Arf6 and the Rho family (RhoA, Cdc42, and Rac1), which induce actin/cytoskeletal rearrangements to promote the shed of ectosomes (Latifkar et al., 2019). External factors can involve in the biogenesis of ectosomes. For example, the influx of calcium induces the redistribution of the phospholipids, thereby promoting the release of ectosomes (Abels and Breakefield, 2016).

### The Cargos of EVs

As a class of highly heterogeneous bilayered structures, EVs carry a rich cargo of various bioactive substances. Firstly, EVs are enriched with a complex of various proteins including cytoskeletal proteins, cytosolic proteins, extracellular matrix proteins, secreted proteins, and plasma membrane proteins such as adhesion proteins, receptors, tetraspanins, transporters, and vesicle trafficking-related proteins (Akbar et al., 2019). Similar to a cell, the lipid membrane of EVs is enriched with cholesterol, ceramide, phosphatidylserine, and sphingolipids (Kreimer et al., 2015). In addition, EVs contain vesicle-specific genetic materials, such as non-coding RNAs (e.g., miRNA, long non-coding RNA, mitochondrial associated tRNA, and small nucleolar RNA), messenger RNAs (mRNAs), and DNAs (e.g., mitochondrial DNA, and even genomic DNA; Stahl and Raposo, 2019). Notably, the composition of EVs can reflect the characteristics of the parental cells. Therefore, analysis of EVs composition showed that some components were specific from cell and tissue of origin, and some are common in all EVs (Kowal et al., 2016). The composition of EVs determines their complexity and potential functional diversity. As EVs carry abundant cargos, many studies have identified the proteomic, transcriptomic, and lipidomic profiles of EVs by high-throughput analysis (Gandham et al., 2020). At the present time, EVs-specific cargos can be accessible on public databases such as ExoCarta, Vesiclepedia, EV-TRACK, and EVpedia (Keerthikumar et al., 2017; Roux et al., 2020). These resources provide insightful information for studying molecular mechanism of EVs’ pathophysiological role underlying different disease conditions.

### The Biologic Function of EVs

Growing evidences indicate that EVs have many cell-intrinsic biologic functions. EVs play important roles in cell-cell communication and exist in various tissues and biological fluids (Sung et al., 2018). EVs can complete intercellular communication in a paracrine or autocrine manner, involving a variety of physiological and pathological responses, such as inflammatory response, invasion and metastasis of tumor cells, angiogenesis, immune response, and drug resistance, etc (Shen et al., 2017).

Due to the characteristics of biocompatibility and stability, EVs can be ideal vehicles for the delivery of drugs, miRNAs, proteins, and other molecules (Villata et al., 2020). Experimental evidence has shown the potential use of EVs in delivering targeted RNA-based therapies in the context of liver diseases (O’Brien et al., 2020). In addition, different EVs contents at different stages can be used as biomarkers and non-invasive diagnostic tools. For instance, circulating miR-135a-3p in serum EVs serve as potential biomarkers of NAFLD (Jiang et al., 2021).

In a word, EVs is promising in clinical applications both as biomarkers and as therapeutic delivery vehicles, but further studies are required to completely resolve the functional capabilities of EVs.

## EVs in the Liver and Upon Alcohol and Lipotoxic Stimulation

Liver is the largest metabolic organ of the human body. The organ is composed of several different types of parenchymal and non-parenchymal cells, which include hepatocytes, biliary epithelial cells (cholangiocytes), Kupffer cells, liver sinusoidal endothelial cells (LSECs), hepatic stellate cells (HSCs), and other immune cells (Trefts et al., 2017). In both physiological and pathological conditions, all types of cells in the liver have been proved to release EVs as well as being targets of endogenous EVs. Compared with the normal physiological condition, the concentration of EVs and the composition of EVs cargos undergo significant changes when the liver was under stress/damage or pathological state (Xie et al., 2019). Healthy human volunteers with binge alcohol drinking or mice after binge or chronic alcohol consumption showed significantly increased number of exosomes in serum. These exosomes were rich in miRNA-122, which is one of the most abundant miRNA in hepatocytes. Alcohol treatment activated the exosome biogenesis pathways by increasing mRNA expression levels of Rab27b, which was critical in controlling late endosomal formation (Momen-Heravi et al., 2015a). A very recent study showed that chronic-plus-binge ethanol induced the production of mtDNA-enriched EVs activation of the ASK1-p38 pathway, which elevated the expression of NSMase2, ALIX, Rab27A protein, and genes related to ESCRT (Hgs, Pdcd61p, Stam1, and Ykt6; Ma et al., 2020). In addition, alcohol also stimulated hepatocytes to release abundant EVs in a caspase-3 activation dependent manner (Verma et al., 2016). Moreover, another study showed that the alcohol-induced increase in EVs generation was associated with disruption of autophagy and impaired autophagosome and lysosome function. This study reveals that alcohol disrupts autophagy function through reduced lysosomal proteins (LAMP1 and LAMP2) in livers with ALD, subsequently leading to a significant increase in EVs release both in macrophages and hepatocytes (Babuta et al., 2019). Alcohol-and-HIV-induced lysosomal dysfunction showed similar effects *in vitro* and in liver-humanized mice (Dagur et al., 2021). These studies clearly illustrated the link between effects of alcohol on EVs biogenesis.

Similar with alcohol exposure, excessive lipid accumulation in hepatocytes was also related with large amounts of EVs release. Lipids can promote EVs release by activating death receptor 5 signaling and ROCK1 (Hirsova et al., 2016). Endoplasmic reticulum (ER) stress was also considered as a key regulator of EVs release in hepatocytes in response to free fatty acid treatment, evidenced by inositol requiring enzyme 1α (IRE1α) deficiency diminished the increased EVs release stimulated by free fatty acid (Kakazu et al., 2016). A new study using *in vivo* NASH model further confirmed the critical role of ER stress in controlling EVs release from liver. High fat, fructose, and cholesterol diet activated IRE1α signaling and EVs generation, while mice with liver specific knockout of IRE1α blocked the elevation of EVs in this model (Dasgupta et al., 2020). In addition to stress, mixed lineage kinase 3 was considered as a key regulator of EVs release in hepatocytes in response to lipotoxicity (Ibrahim et al., 2016).

As mentioned above, the composition of EVs changes significantly under stress/injury or pathological conditions. Alcohol and/or its metabolites can increase the amounts of EVs cargos. For instance, the amount of CYP2E1, other P450 isoforms and some hepatic proteins were elevated in EVs released from alcohol exposed liver (Cho et al., 2017b). Similarly, hepatocyte abundant miR-122 and miR-192 were enriched in EVs isolated from serum of mice with fatty liver (Povero et al., 2014).

## EVs in the Pathogenesis of ALD and NAFLD

### EVs in the Development of ALD

Alcohol and its metabolites can induce a wide spectrum of hepatic lesions, the most common characteristic of which are fatty liver, hepatic inflammation, and fibrosis/cirrhosis (Dunn and Shah, 2016). Previous studies have identified several mechanisms in the progression of ALD, such as acetaldehyde and oxidative stress related to ethanol metabolism, epigenetic changes, ethanol-mediated induction of endotoxins from the impairment of intestinal permeability and subsequent activation of Kupffer cells (Seitz et al., 2018). In addition, second hits include the complex of pharmacological, genetic, and viral factors, which can promote the progression of ALD. Currently, the contribution of EVs in the pathogenesis and progression of ALD is under evaluation. Research evidence suggests that the levels of CYP2E1 and other P450 isoforms are elevated in circulating EVs from alcohol-exposed mice and patients with alcoholism. EVs containing CYP2E1 and other P450 isoforms can further promote hepatocyte death by activating apoptosis signaling pathways (Cho et al., 2017b). Recent data indicate that long-term excessive intake of ethanol can promote the release of EVs rich in pro-inflammatory mitochondrial DNA (mtDNA) by hepatocytes. These mtDNA enriched EVs were response for neutrophil recruitment and activation in the liver, which further damage the liver (Cai et al., 2017; Ma et al., 2020).

Hepatocytes derived EVs can also impact other cells in the liver, especially liver macrophages. EVs isolated from serum of patients with alcohol binge drinking or *in vitro* ethanol-treated hepatocytes were taken up by recipient monocytes\macrophages, and the mature form of liver-specific miRNA-122 was transferred horizontally. EVs-mediated high levels of miRNA-122 can increase the sensitivity of monocytes to LPS, thereby inducing more pro-inflammatory cytokines (Momen-Heravi et al., 2015a). In addition, hepatocyte-derived EVs activated macrophages into an M1 inflammatory phenotype. The CD40L contained in hepatocyte-derived EVs contributed to the activation of pro-inflammatory macrophage associated with alcohol-induced liver damage (Verma et al., 2016). Another report showed that the number of circulating EVs was increased in ALD mice. The ALD associated EVs can be uptake by both hepatocytes and macrophages, which enhanced monocyte chemoattractant protein 1 expression and increased the number of M1 Kupffer cells/infiltrated monocytes. The effects of ALD EVs on liver macrophages was mediated by abundant Heat shock protein 90 (Hsp90) in ALD EVs (Saha et al., 2018). Notably, hepatocyte-derived EVs release were increased in AH and these EVs promote HSCs activation by specific miRNAs (e.g., miR-27a and miR-181a), which can target and repress the quiescent HSCs marker. In addition, AH-hepatocyte-derived EVs were enriched with DAMPs (e.g., mtDNA), which subsequently activated hepatic macrophages through toll-like receptor 9 (TLR-9) signaling (Eguchi et al., 2020).

Besides hepatocytes, monocyte/macrophage can also secrete more EVs after alcohol exposures. It was shown that alcohol dose-dependently increased EVs production in monocytes. EVs derived from alcohol treated monocytes promoted the M2 differentiation of naïve recipient monocytes. miR-27a was identified as the major contributor of the effects of EVs derived from alcohol treated monocytes (Saha et al., 2016).

Extracellular vesicles also played an important role in the process of second hits aggravating ALD. For instance, the human plasma-derived exosomes contain substantial levels of metabolically active CYP2E1, which play a key role in exacerbating alcohol and acetaminophen-(APAP) induced toxicity in hepatocytes (Rahman et al., 2019). Another study showed that some cytokines and chemokines, such as IL-8, IL-10, and IL-1ra were found specifically packaged in EVs from HIV-infected alcohol drinkers, which implied a novel mechanism for the involvement of EVs in the development of ALD (Kodidela et al., 2018).

**Figure 2:**
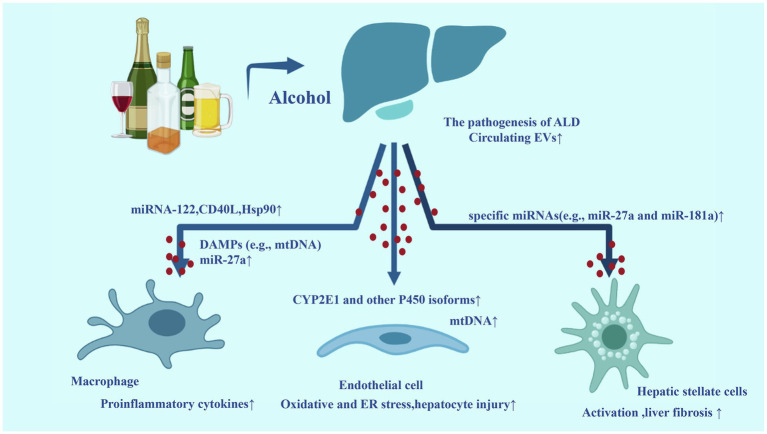
Extracellular vesicles in the development of alcoholic liver disease (ALD). Alcohol exposure in the liver can increase the number of circulating EVs. Some biomolecules loaded by EVs are upregulated, acting on target cells [such as macrophages, endothelial cells, and hepatic stellate cells (HSCs)] and promoting inflammation and fibrosis. The release of circulating EVs containing a high level of these biomolecules is related to the progression of ALD to their inflammatory and more aggressive forms alcoholic steatohepatitis (ASH).

All these studies have established solid evidences for the key role of EVs in the development of ALD. We have summarized these mechanisms in Figure 2.

### EVs in the Development of NAFLD

Certain toxic lipids such as saturated free fatty acids, free cholesterol, or ceramides and other sphingolipids accumulate in hepatocytes during the progression of NAFLD, leading to hepatocyte stress/injury through lipotoxicity. EVs are produced in large quantities during lipotoxicity and are involved in key processes in the pathogenesis of NAFLD, including angiogenesis, fibrosis, and inflammation. Hepatocytes are the main cell type of the liver, accounting for 80% of the liver volume. Studies on mouse models and human subjects with NASH showed that hepatocytes are the main contributors to the elevated levels of circulating EVs (Srinivas et al., 2020). Therefore, hepatocyte-derived EVs may play a key role in the pathogenesis and progression of NAFLD. For example, lipotoxic hepatocyte-derived EVs can promote liver inflammation through the interaction with macrophages, which express and release of several proinflammatory cytokines, such as IL-1β and IL-6. These EVs have been shown to contain TRAIL, which induce macrophage activation by activating NF-κB pathway (Hirsova et al., 2016). Palmitate as a precursor for some toxic lipid moieties, such as lysophosphatidyl choline, can induce the release of C16:0 ceramide-enriched EVs. The ceramide metabolites contain sphingosine-1-phosphate (S1P), which promoted macrophage recruitment to the liver *via* S1P1 receptor mediated macrophage chemotaxis and enhanced liver inflammation (Kakazu et al., 2016). In Steatohepatitis, activated IRE1A-induced the massive release of EVs, thereby recruiting macrophages into the liver and promoting liver inflammation by transmitting hepatocellular ER stress to macrophages (Dasgupta et al., 2020). Additionally, lipotoxic hepatocyte-derived EVs were enriched with the (C-X-C motif) ligand 10 (CXCL10), which was a potent chemokine for macrophage chemotaxis. EVs-associated CXCL10 was more effective than free chemokines in stimulating macrophage chemotaxis (Ibrahim et al., 2016). Another study showed that increased circulating EVs in NASH were enriched with mtDNA, which subsequently activated macrophage by TLR-9 inflammatory signal (Garcia-Martinez et al., 2016).

The progression of NAFLD is significantly associated with the increase of visceral adiposity. EVs from visceral adipose tissue can target hepatocyte and HSC lines *in vitro*. These EVs increased matrix metalloproteinase-1 (TIMP-1) and integrin ανβ-5 expression and decreased matrix metalloproteinase (MMP)-7 and plasminogen activator inhibitor-1 expression in hepatocytes, while increased expression of TIMP-1, TIMP-4, Smad-3, integrin ανβ-5, integrin ανβ-8, and MMP-9 in HSCs. These changes caused dysregulation of TGFβ signaling pathway and may contribute to liver fibrosis in NASH (Koeck et al., 2014).

Lipotoxic hepatocyte derived EVs can be efficiently uptaken by HSCs. These EVs are rich in miR-128-3p, which inhibits peroxisome proliferator-activated receptor-γ (PPAR-γ) and consequent phenotypical switch from quiescent to activated HSCs (Povero et al., 2015). The angiogenesis is a key pathological feature in the progression to NASH, particularly the development of fibrosis. Hepatocyte-derived EVs can as proangiogenic factor and induce endothelial cell migration and tube formation *in vitro* and angiogenesis *in vivo* in a process requiring VNN1-dependent internalization (Povero et al., 2013).

Hepatic non-parenchymal cell-derived EVs are also contributed to the pathogenesis and progression of NAFLD. For instance, activated HSCs-derived EVs mediated its intercellular transfer to other quiescent or activated HSCs and these EVs were enriched with the connective tissue growth factor (CCN2), which may cause enhancement or fine tuning of fibrogenic signaling in response to chronic liver injury (Charrier et al., 2014). Endothelial cell-derived EVs could regulate the phenotype and migration of HSCs *via* transfer of sphingosine kinase 1 (SK1) and its product S1P (Wang et al., 2015).

**Figure 3:**
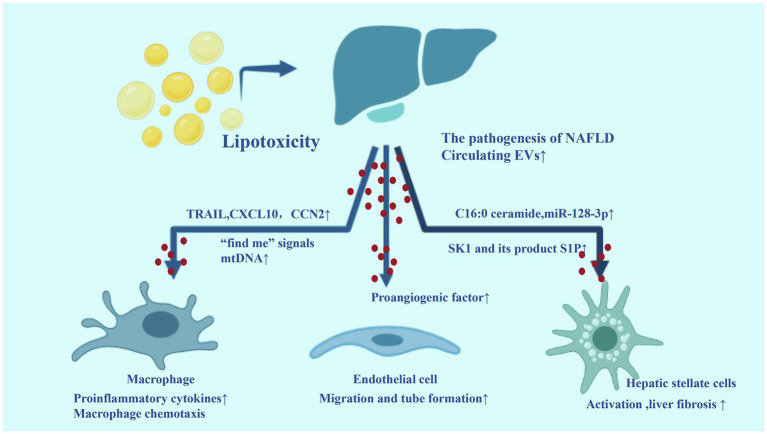
Extracellular vesicles in the development of non-alcoholic fatty liver disease (NAFLD). During the process of lipotoxicity, a large number of EVs released from liver carry inflammatory molecules to liver non-parenchymal cells (macrophages, HSCs, and endothelial cell), which can promote liver inflammation, angiogenesis, and fibrosis.

Taken together, EVs released from stressed/injured hepatocytes target liver non-parenchymal cells such as macrophages and HSCs, linking lipotoxicity with events such as inflammation and fibrosis, which are associated with the progression of NAFLD to NASH. In addition, hepatic non-parenchymal cell-derived EVs are also contributed to the development of NAFLD. We have summarized the EVs in NASH in Figure 3.

## The Therapeutic and Diagnostic Potential of EVs in NAFLD and ALD

### EVs Based Therapeutics for NAFLD and ALD

From the perspective of disease treatment, unmodified or engineered EVs can be used as therapeutics for liver diseases. The therapeutic potential of EVs has become a hot research topic recently (Murphy et al., 2019). Due to the inherent regenerative capacity of liver tissue and the tropism of systemic injection of nanovesicles in this organ, the liver is an ideal target for EVs based therapy (Borrelli et al., 2018). Currently, EVs based therapy on liver diseases are still in preclinical stage and in most of these studies, EVs are derived from mesenchymal stem cells (MSCs; Driscoll et al., 2021). EVs from amnion-derived MSCs may improve inflammation and fibrogenesis in rat models of NASH and liver fibrosis by attenuating the activation of HSC and Kupffer cells (Ohara et al., 2018). EVs form human liver stem cells showed similar beneficial effects in mouse model of NASH (Bruno et al., 2020b). Our own study confirmed that myeloid-specific IL-6 induces myeloid cells to release miR-223-enriched EVs, which are then targeted to hepatocytes to attenuate NAFLD-associated fibrosis (Hou et al., 2020). In addition, induced pluripotent stem cells (iPSC)-derived EVs could be internalized by HSCs and exerted anti-fibrotic effects. These iPSC-EVs contained several specific miRNAs (such as miR-92a-3p and miR-302-3p), which can inhibit fibrogenesis (Povero et al., 2019). These are unmodified EVs directly used as therapeutic agents. In terms of ALD treatment, EVs based therapy has not been extensively studied. There were some clues that using small molecule compounds to regulate biogenesis and release EVs may benefit ALD. For instance, CYP2E1 inhibitor chlormethiazole efficiently blocked the elevated EVs release from hepatocytes in response to alcohol (Cho et al., 2018).

Most of engineered EVs use different methods to load the required goods such as specific biomolecules and small molecule drugs into EVs for targeted therapy (Wu et al., 2021). In terms of EVs as vehicles for small molecule drugs, EVs-mediated miRNA-122 inhibitors use RNA interference to prevent the pro-inflammatory EVs produced by alcohol-treated hepatocytes (Momen-Heravi et al., 2015a). However, the number of studies on this treatment is quite limited in the areas of NAFLD.

Extracellular vesicles have broad prospects in the therapeutic field. They have the ability to overcome natural obstacles, their inherent cell targeting properties, and their stability in the circulation (Vader et al., 2016). However, EVs-based therapeutics face several remaining challenges, such as large-scale EVs production and safety issue (Bruno et al., 2020a). Therefore, due to these current significant limitations, there is still a long way to go for EVs to have clinical translational utility.

### EVs as Biomarkers for NAFLD and ALD

As mentioned above, the total number of circulating EVs was significantly increased in patients with various liver diseases. Based on the stability of EVs, it exists in most body fluids and iconic cargoes such as nucleic acid and protein, so it has the potential to be a circulating biomarker (Szabo and Momen-Heravi, 2017). Numerous studies have therefore investigated the potential applications of EVs-based diagnostic markers in the field of NAFLD and ALD. For instance, CD14+ macrophage/monocytes and iNKT derived EVs can be used as markers of the severity of liver injury and fibrosis in NAFLD patients (Kornek et al., 2012). Serum EVs miR-192-5p can be used as a potential noninvasive biomarker for disease progression of NAFLD (Liu et al., 2020). Additionally, EVs miR-122 and miR-192 can as a biomarker for the diagnosis of advanced NAFLD or NASH in serum from NAFLD patients (Lee et al., 2017). For ALD, circulating miRNAs in EVs can be used to distinguish liver injury in different mouse models including ALD, drug induced liver injury and inflammatory liver damage (Bala et al., 2012). Furthermore, in human alcoholic hepatitis (AH) patients, miRNA-192 and miRNA-30a in EVs were significantly increased in the plasma (Momen-Heravi et al., 2015b). The APAP- and ethanol-induced liver injury increased the amounts of liver-specific proteins (e.g., GST, ALB, HP, and FGB) in circulating EVs, which can served as reliable and noninvasive biomarkers (Cho et al., 2017a). Notably, the latest research shows that the number of circulating EVs and their sphingolipid cargoes can be used very powerful biomarker to distinguish AH from heavy drinkers, decompensated alcohol-associated cirrhosis and other end-stage liver diseases. The incorporation of circulating EVs concentration and sphingolipid cargo with traditional Model for End Stage Liver Disease score provided a better performance in predicting 90-day mortality of AH patients (Sehrawat et al., 2021). These findings indicated that EVs together with other biomarkers can potentially serve as a more powerful and accurate tool for diagnosis.

## Conclusion

In recent years, EVs in the field of metabolic liver disease has gathered significant traction. During lipotoxicity and alcohol exposure, EVs are released in large quantities through different mechanisms. These EVs plays a key role in the transmission of information between different cell types in the liver. They can change the function of target cells and activate different pathways, thereby promoting the occurrence, pathogenesis and development of NAFLD and ALD. Understanding these issues will help us to understand the mechanism of disease and study potential therapeutic interventions. EVs may provide new clues for the therapeutic and diagnosis, but this field is still in its infancy. With more research on EVs, it is believed that EVs can be successfully used in the treatment and clinical diagnosis of NAFLD/ALD in the near future.

## Author Contributions

DW, HZ, and HW contributed to conception and design of the work. DW wrote the first draft of the manuscript. HZ and HW wrote the sections of the manuscript. All authors contributed to the article and approved the submitted version.

### Conflict of Interest

The authors declare that the research was conducted in the absence of any commercial or financial relationships that could be construed as a potential conflict of interest.
